# Relationship Between Bacterial Strain Type, Host Biomarkers, and Mortality in *Clostridium difficile* Infection

**DOI:** 10.1093/cid/cit127

**Published:** 2013-03-05

**Authors:** A. Sarah Walker, David W. Eyre, David H. Wyllie, Kate E. Dingle, David Griffiths, Brian Shine, Sarah Oakley, Lily O'Connor, John Finney, Alison Vaughan, Derrick W. Crook, Mark H. Wilcox, Tim E. A. Peto, on behalf of the Infections in Oxfordshire Research Database (IORD)

**Affiliations:** 1 NIHR Biomedical Research Centre, Oxford; 2 Nuffield Department of Medicine; 3 Nuffield Department of Clinical Laboratory Sciences, University of Oxford; 4 MRC Clinical Trials Unit, London; 5 Oxford University Hospitals Trust, Oxford; 6 Leeds Institute of Molecular Medicine, University of Leeds, United Kingdom

**Keywords:** *C. difficile*, mortality, biomarkers, strain-specific variation

## Abstract

***Background.*** Despite substantial interest in biomarkers, their impact on clinical outcomes and variation with bacterial strain has rarely been explored using integrated databases.

***Methods.*** From September 2006 to May 2011, strains isolated from *Clostridium difficile* toxin enzyme immunoassay (EIA)–positive fecal samples from Oxfordshire, United Kingdom (approximately 600 000 people) underwent multilocus sequence typing. Fourteen-day mortality and levels of 15 baseline biomarkers were compared between consecutive *C. difficile* infections (CDIs) from different clades/sequence types (STs) and EIA-negative controls using Cox and normal regression adjusted for demographic/clinical factors.

***Results.*** Fourteen-day mortality was 13% in 2222 adults with 2745 EIA-positive samples (median, 78 years) vs 5% in 20 722 adults with 27 550 EIA-negative samples (median, 74 years) (absolute attributable mortality, 7.7%; 95% CI, 6.4%–9.0%). Mortality was highest in clade 5 CDIs (25% [16 of 63]; polymerase chain reaction (PCR) ribotype 078/ST 11), then clade 2 (20% [111 of 560]; 99% PCR ribotype 027/ST 1) versus clade 1 (12% [137 of 1168]; adjusted *P* < .0001). Within clade 1, 14-day mortality was only 4% (3 of 84) in ST 44 (PCR ribotype 015) (adjusted *P* = .05 vs other clade 1). Mean baseline neutrophil counts also varied significantly by genotype: 12.4, 11.6, and 9.5 × 10^9^ neutrophils/L for clades 5, 2 and 1, respectively, vs 7.0 × 10^9^ neutrophils/L in EIA-negative controls (*P* < .0001) and 7.9 × 10^9^ neutrophils/L in ST 44 (*P* = .08). There were strong associations between *C. difficile*-type-specific effects on mortality and neutrophil/white cell counts (rho = 0.48), C-reactive-protein (rho = 0.43), eosinophil counts (rho = −0.45), and serum albumin (rho = −0.47). Biomarkers predicted 30%–40% of clade-specific mortality differences.

***Conclusions.*** *C. difficile* genotype predicts mortality, and excess mortality correlates with genotype-specific changes in biomarkers, strongly implicating inflammatory pathways as a major influence on poor outcome after CDI. PCR ribotype 078/ST 11 (clade 5) leads to severe CDI; thus ongoing surveillance remains essential.

## Abstract (precis)

*Clostridium difficile* genotype predicts 14-day mortality in 1893 enzyme immunoassay–positive/culture-positive adults. Excess mortality correlates with genotype-specific changes in biomarkers, strongly implicating inflammatory pathways as a major influence on poor outcome. Polymerase chain reaction ribotype 078/ST 11(clade 5) is associated with high mortality; ongoing surveillance remains essential.

**(See the Editorial Commentary by Gerding and Johnson on pages 1601–3.)**

The widespread emergence of hypervirulent polymerase chain reaction (PCR) ribotype 027/NAP1/BI/sequence type (ST) 1 [1] strains in the early 2000s [2, 3] substantially increased *Clostridium difficile* infection (CDI) incidence. PCR ribotype 027 has also been associated with more severe outcomes in most [2, 4, 5] but not all [6–9] studies. Outcome variation across non-027 strains has rarely been investigated, invariably with small numbers, although these now account for most new CDIs. One study [6] (n = 395) found significantly more complicated disease outcomes with PCR ribotypes 018 (ST 17 from [10]; n = 23) and 056 (ST 34/58 [10]; n = 6), whereas another [11] (n = 168) reported similar 30-day mortality in PCR ribotype-027 (n = 46) and 017 (ST 37 [10]; n = 57). Although PCR ribotype 078 (ST 11), common in livestock [12] and rising in incidence [6, 13], is denoted hypervirulent on the basis of increased toxin production [14] and individual case severity [15], supporting clinical data are few. Attributable mortality and severe diarrhea was similar in PCR ribotype 078 (n = 54) and 027 (n = 124) in 1 study (both greater than in 501 non-027/078 cases) [13], but PCR ribotype 078 (n = 31) was not associated with complicated CDI in another [6]. Although scores to predict CDI severity, complications, or recurrence have variably included biomarkers (eg, white blood count [WBC], C-reactive protein [CRP]) [16], no studies have investigated associations between CDI strains and biomarkers.

We aimed therefore to investigate whether the genotype of *C. difficile* clinical isolates from multilocus sequence typing (MLST) was associated with mortality and severity biomarkers using a large population-based database of CDI cases and to explore associations between strain-specific effects on host biomarkers and mortality to provide insights into infection pathogenesis.

## 

## METHODS

Oxford University Hospitals (OUH) NHS Trust provides >90% of hospital care and all acute services in Oxfordshire (approximately 600 000 people). It includes 2 large acute teaching hospitals and 1 specialist orthopedic hospital in Oxford and 1 district hospital 35 miles north. The OUH microbiology laboratory tests all stool samples from the county, including those from other healthcare facilities/primary care. From 12 September 2006 to 21 May 2011, all unformed stools submitted for *C. difficile* toxin testing, positive by enzyme immunoassay (EIA) and with sufficient sample remaining, were routinely cultured and MLST typed [1]. During this period, infection control policy required all inpatients with diarrhea (≥3 unformed stools within 24 hours) to have samples sent for EIA testing and to initiate vancomycin treatment empirically, continuing for 14 days if CDI was confirmed. Additionally, from May 2007, all unformed samples from those aged ≥65 years were routinely EIA tested following UK policy.

*C. difficile* MLST data were anonymously linked to OUH hospital admissions/discharges, mortality, and laboratory test results from the Infections in Oxfordshire Research Database (IORD) through 21 August 2011 [17]. Admissions to other much smaller regional (including psychiatric/community) hospitals were not included, although samples taken at these locations were identifiable. Rates were calculated using overnight stays defined by the UK KH03 occupancy statistic. IORD has Research Ethics Committee (09/H0606/85) and UK National Information Governance Board (5-07(a)/2009) approval as an anonymized database without individual informed consent.

The primary outcome was 14-day mortality after EIA-based CDI detection in adults aged ≥18 years (excluding repeat EIA-positive cases within 14 days; censoring follow-up at 14 days). EIA-negative samples were included as controls (excluding repeat negatives within 14 days and any sample taken after or within 21 days before the first EIA positive). See Supplementary Material for details.

The primary exposure was type of CDI, categorized by EIA/culture status or *C. difficile* phylogenetic clade from MLST [1]. CDI-associated MLST STs correlate reasonably closely with ribotype [18] and can be grouped by evolutionary relationships into clades [10]. These clades persist despite homologous recombination and have the same phylogenetic structure with MLST or whole-genome sequences [19], suggesting they may behave more similarly in humans. Adjusted mortality risks in each clade and STs with >20 cases were estimated using Cox models, with robust variance adjustment for multiple episodes per patient [20]. EIA-negative controls comprised the reference category so that risks reflected CDI-attributable mortality. Independent predictors were identified using backward selection with the Akaike information criterion [21], allowing nonlinear effects of continuous factors [22]. Exposures considered were demographics, sample characteristics, previous hospital exposure, and previous healthcare-associated infections (Table 1) (antibiotic exposure not available). The impact of clade on the 15 biomarkers available for >50% cases within −3 to +1 days of sample collection was estimated using normal regression on BoxCox-transformed values. Associations between biomarkers and 14-day mortality were estimated using Cox models with multiple imputation (see Supplementary Material).

**Table 1.** Characteristics of *Clostridium difficile* Samples 12 September 2006–21 May 2011 and Relationship With 14-Day Mortality

		Number (%) or Median (IQR)		Unadjusted Univariable Model			Adjusted Multivariable Model^a^		
Factor	Levels (Effect in Cox Model)	In EIA Negative Controls	In EIA Positive Cases	HR	(95% CI)	*P*	HR	(95% CI)	*P*
Type of test	EIA negative	27 550 (100%)	…	1.00		<.0001	1.00		<.0001
	EIA positive/culture negative		571 (21%)	1.59	(1.19–2.12)		1.59	(.93–2.73)	
	EIA positive/not cultured		281 (10%)	2.61	(1.89–3.61)		2.45	(1.62–3.70)	
	Clade 1		1168 (43%)	2.23	(1.88–2.66)		2.32	(1.71–3.13)	
	Clade 2 (027/ST 1)		560 (20%)	3.95	(3.26–4.79)		3.40	(2.45–4.68)	
	Clade 3 (023)		73 (3%)	1.31	(.53–3.26)		1.65	(.62–4.36)	
	Clade 4 (017/ST 37)		29 (1%)	2.74	(1.04–7.21)		2.65	(.99–7.13)	
	Clade 5 (078/ST 11)		63 (2%)	5.17	(3.16–8.46)		5.37	(3.10–9.32)	
Demographics									
Sex	Female (vs male)	15 682 (57%)	1566 (57%)	0.79	(.72–.86)	<.0001	0.75	(.68–.82)	<.0001
Age, years	Per 10 years older	74 (63–83)	78 (67–85)	1.42	(1.37–1.47)	<.0001	1.41^b^	(1.36–1.47)	<.0001
Sample characteristics									
Location where sample taken	Inpatient	16 598 (60%)	1860 (68%)	1.00		<.0001	1.00		<.0001
	Primary care	8108 (29%)	557 (20%)	0.14	(.12–.17)		0.06^c^	(.03–.14)	
	Outpatient/ER/day case	1395 (5%)	148 (5%)	0.35	(.27–.47)		0.98^c^	(.35–2.78)	
	Other hospital	1449 (5%)	180 (7%)	0.50	(.40–.63)		0.12^c^	(.05–.30)	
If inpatient, speciality	Surgical	6112 (37%)	549 (30%)	1.00		<.0001	1.00		<.0001
	Medical	10 486 (63%)	1311 (70%)	1.91	(1.71–2.15)		1.64	(1.44–1.88) if EIA−	
							1.64	(.88–3.06) if EIA +, cult −	
							0.98	(.73–1.30) if EIA +, cult + (interaction *P* = .004)	
If inpatient, method	Elective	3609 (22%)	363 (20%)	1.00		<.0001	1.00		.01
	Emergency	12 989 (78%)	1497 (80%)	1.64	(1.43–1.88)	<.0001	1.22	(1.04–1.43)	
If inpatient, days since admitted	Nonlinear effect^d^	5 (2–12)	9 (2–22)			<.0001			<.0001
	(Days/10)^−1^			0.87	(.78–.97)		0.76*^d^*	(.68–.84)	
	ln(days/10)^a^(days/10)^−1^			1.00	(.95–1.04)		0.90*^d^*	(.86–.94)	
Clinician requested EIA test when submitting sample	No (mild diarrhea) (vs yes)	7895 (29%)	436 (16%)	0.48	(.42–.54)	<.0001	0.69	(.51–.92)	.01
Days since last negative EIA test^e^	(For every day closer in the last 2 wk)	…	4 (1–8) (if test in last 2 wk)	0.97^e^	(.95–1.00)	.02	0.96	(.94–.99)	.007
Previous *C. difficile*	Yes (vs no)	0 (0%)	634 (23%)	0.99^e^	(.78–1.26)	.94	*(p* = *0.18)*		
Previous hospital exposure (strictly before the current admission, if inpatient)									
Ever previously admitted to OUH	Yes, for ≥1 admission >8 hours	19 570 (71%)	2253 (82%)	1.00		<.0001	1.00		.01
	Yes, but only for <8 hour admissions	2462 (9%)	139 (5%)	0.55	(.45–.68)		0.93	(.71–1.21)	
	Never	5518 (20%)	353 (13%)	0.63	(.55–.72)		1.30	(1.03–1.63)	
Previously admitted to GI ward	Yes (vs no)	8484 (31%)	981 (36%)	0.95	(.86–1.05)	.34	0.89	(.80–.99)	.03
Dialysis/chemotherapy at OUH	Yes (vs no)	3051 (11%)	332 (12%)	1.37	(1.21–1.56)	<.0001	1.39	(1.21–1.60)	<.0001
Number of previous admissions >8 hours	(per 5 additional >8 hours admissions)	2 (1–4)	2 (1–5)	1.06^f^	(.99–1.12)	.08	0.92	(.84–1.00)	.06
Previous hospital stay (hours)	(Per doubling of total previous hours in hospital)	169 (8–656)	478 (77–1229)	1.11^g^	(1.09–1.13)	<.0001	1.02^g^	(.99–1.06)	.20
Days since last discharged	(Per additional 6 mo since last OUH discharge)	285 (42 to >1096)	78 (22–640)	0.92	(.90–.95)	<.0001	0.96	(.93–.98)	.002
SHEA [35] classification									
	HO-HCFA	11 628 (42%)	1373 (50%)	1.00		<.0001	(*P* = .93)		
	CO-HCFA	3432 (12%)	604 (22%)	0.66	(.57–.76)				
	Indeterminate	1892 (7%)	248 (9%)	0.54	(.45–.66)				
	CO	10 598 (38%)	520 (19%)	0.30	(.26–.34)				

Abbreviations: CI, confidence interval; CO, community onset; CO-HCFA, community onset–health-care facility associated; cult, culture; EIA, enzyme immunoassay; ER, emergency room; GI, gastrointestinal; HO-HCFA, hospital onset–health-care facility associated; HR, hazard ratio; IQR, interquartile range; OUH, Oxford University Hospitals; SHEA, Society for Healthcare Epidemiology of America

^a^ HR with opposite effect to unadjusted univariable models due to confounding are underlined. *P* values in italics show the nonsignificant effects of adding in factors not chosen by the Akaike information criterion selection.

^b^ Although mortality was lower after tests that had not been directly requested by the clinician, the increase in risk with age was significantly greater following these tests (per 10 years HR = 1.71; 95% CI, 1.48–1.98; interaction *P* = .009). For those aged <84.4 years, mortality risks were therefore greater after clinician-requested tests; fore those aged ≥84.4 years, mortality risks were greater after tests that had not originally been requested by the clinician.

^c^ Mortality reduced even further if EIA test is negative rather than positive (additional HR = 0.63; 95% CI, .43–.94; *P* = .02).

^d^ Significant nonlinearity, with greatest risk of death on day of admission, then dropping sharply, and then gradually rising.

^e^ Univariable model also adjusts for positive vs negative EIA test.

^f^ Univariable model also adjusts for ever vs never previously admitted.

^g^ Effects significantly (*P* < .0001) stronger if samples taken in primary care (HR = 1.25; 95% CI, 1.16–1.36 per doubling) or other hospitals (HR = 1.27; 95% CI, 1.16–1.39 per doubling) than as inpatients (HR in table above) or outpatients/ER/day cases (HR = 0.98; 95% CI, .88–1.10 per doubling; interaction *P* < .0001).

## RESULTS

**Figure 1.**
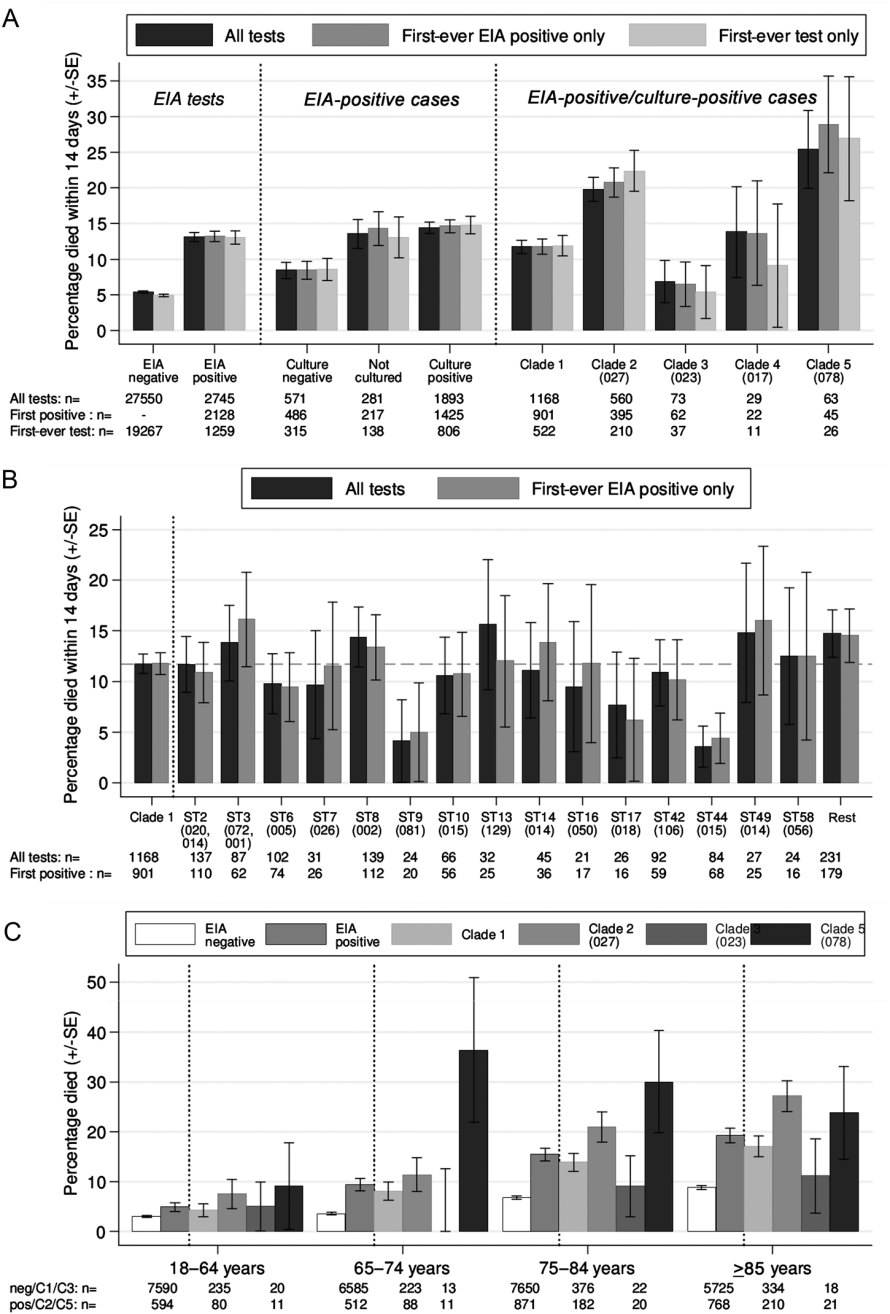
Fourteen-day mortality after enzyme immunoassay (EIA) tests for *Clostridium difficile*, overall and by strain. *A*, Fourteen-day mortality by EIA-negative control vs EIA-positive case and multilocus sequencing type clade if culture positive. *B*, Fourteen-day mortality by sequence type within clade 1. *C*, Fourteen-day mortality by age (all tests). Most common ribotypes of isolates from each clade (*A*) or sequence type (B) shown in brackets. Dashed line in (*B*) shows overall clade 1 mortality. Clade 4 not shown in (*C*) due to small numbers (n = 29). Abbreviations: EIA, enzyme immunoassay.

From September 2006 to May 2011, after 14-day deduplication, there were 2745 consecutive toxin-EIA-positive stools in 2222 adults (median age, 78 years; interquartile range [IQR], 67–85 years; 2128 (78%) first ever EIA-positive) and 27 550 consecutive EIA-negative stools in 20 722 adults without a previous positive (median age, 74 years; IQR, 63–83 years). Crude 14-day mortality was similar after first (13%) vs subsequent (13%) EIA-positive cases and first (5%) vs subsequent (7%) EIA-negative controls (Figure 1*A*). Overall attributable mortality was 7.7% (95% confidence interval [CI], 6.4%–9.0%; *P* < .0001; Figure 1*A*). Fourteen-day mortality was lower after EIA-positive/culture-negative cases (8%) than after EIA-positive/culture-positive cases (14%; *P* < .0001), although still higher than the 5% in EIA-negative/culture-negative controls (*P* = .002).

**Figure 2.**
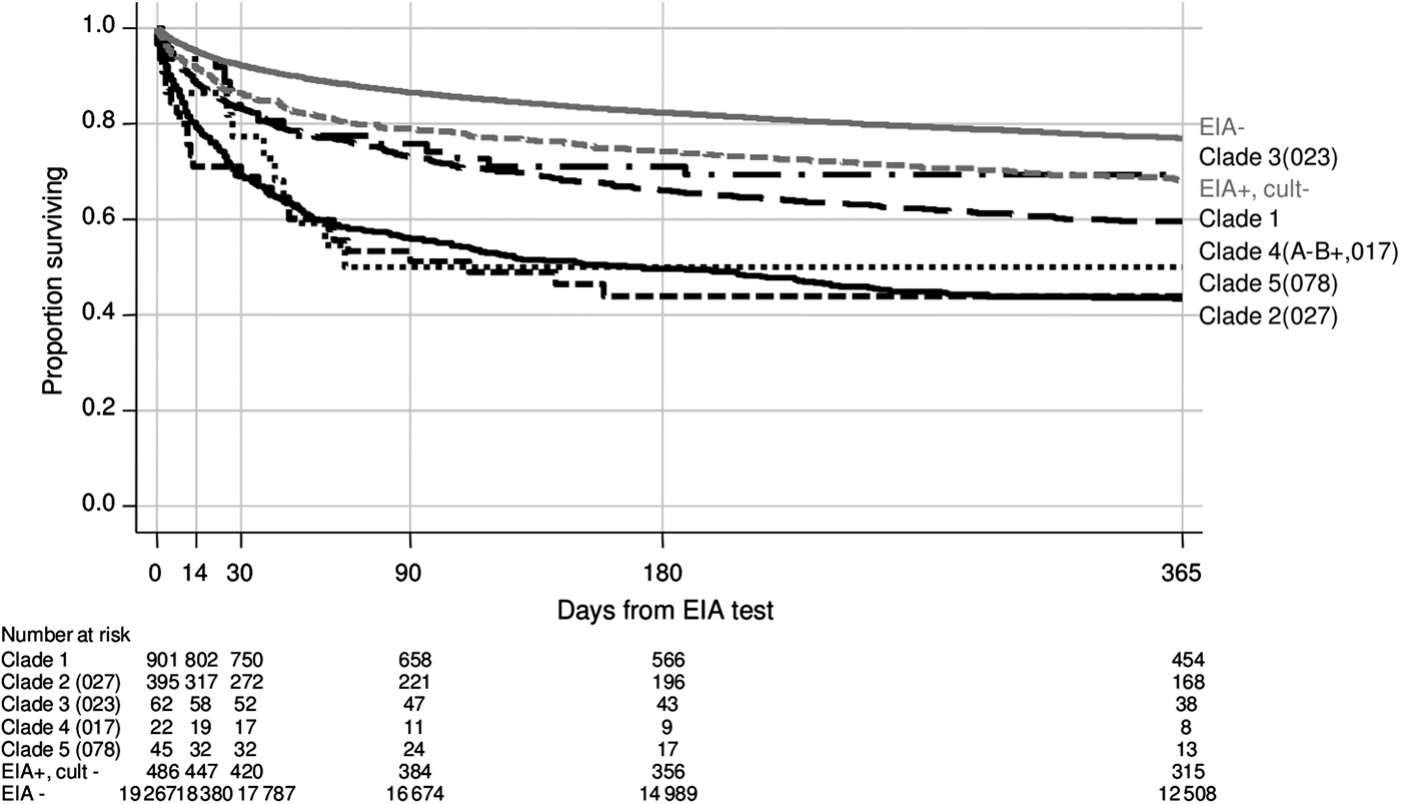
One-year mortality after first-ever *Clostridium difficile* enzyme immunoassay–positive test or first negative before positive test by strain. Abbreviation: EIA, enzyme immunoassay.

In EIA-positive/culture-positive cases, there were substantial mortality differences between *C. difficile* clades (*P* < .0001; Figure 1*A*). Fourteen-day mortality was highest in clade 5 (25%; all PCR ribotype 078/ST 11 [10]), then clade 2 (20%; 99% PCR ribotype 027/ST 1), clade 4 (14%; 97% A-B+ PCR ribotype 017/ST 37), and clade 1 (12%); lowest mortality occurred in clade 3 (7%; all PCR ribotype 023). The heterogeneous clade 1 had 67 STs, 15 with >20 isolates. Observed mortality varied markedly between common clade 1 STs (median, 11%; range, 4%–16%; Figure 1*B*), although small numbers limited power to distinguish genuine from chance differences (exact *P* = .76). Fourteen-day mortality was only 4% in ST 44 (95% CI, .7%–10%; exact *P* = .01 vs other clade 1, post hoc test). Similar relative differences between clades were observed at all ages (Figure 1*C*). Over the longer term, mortality was consistently higher in clades 2 and 5 and lower in clades 1 and 3 (Figure 2). In inpatients not dying before 14 days, the median stay post–EIA test was significantly longer in EIA-positive cases (median, 16; IQR, 7–32) than in EIA-negative controls (median, 9; IQR, 3–21; *P* = .0001) and in clade 2 (median, 19; IQR, 10–34) vs 1 (median, 15; IQR 7–32; *P* = .005).

**Figure 3.**
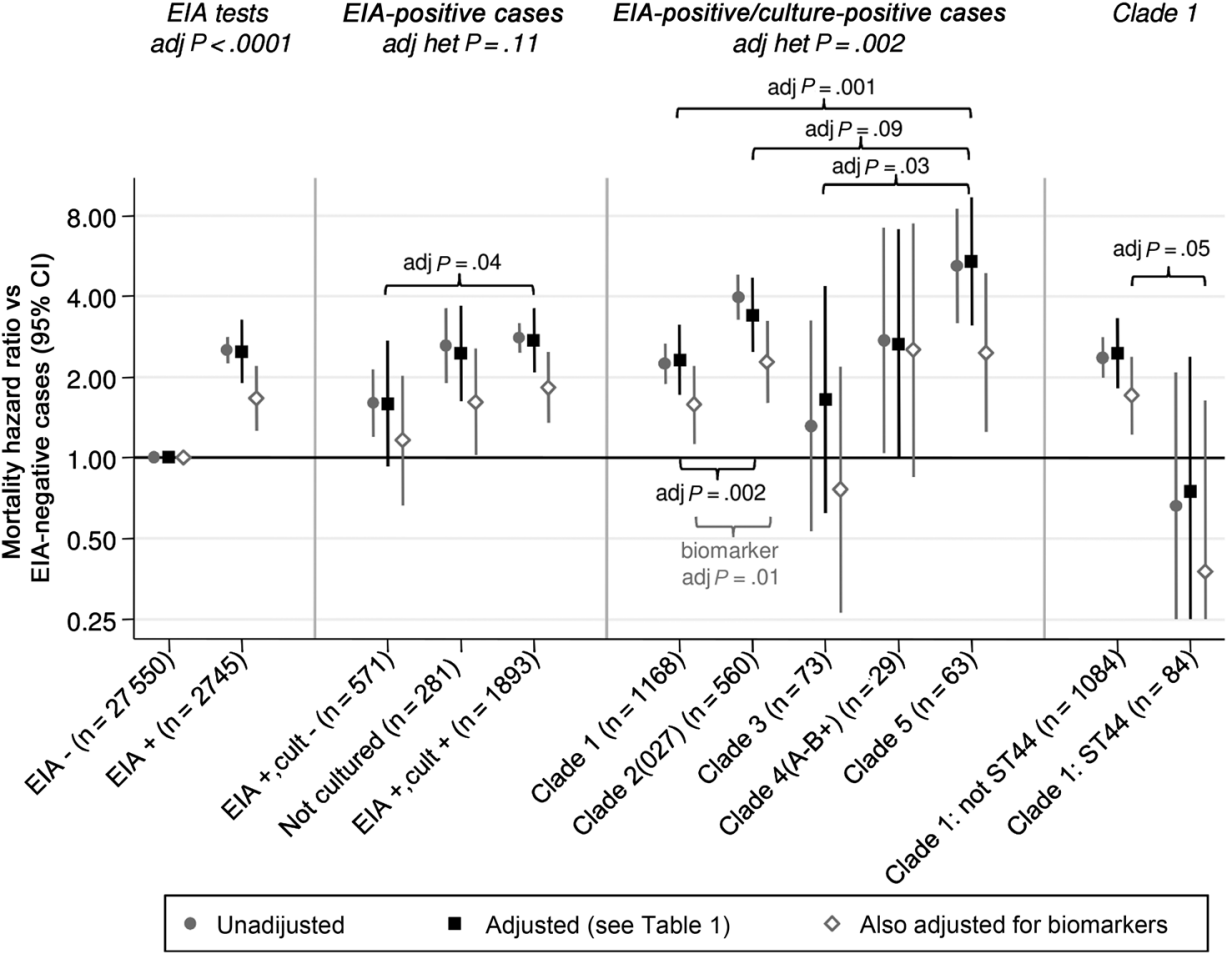
Variation in 14-day mortality risks according to *Clostridium difficile* clade. Abbreviations: adj, adjusted; CI, confidence interval; cult, culture; EIA, enzyme immunoassay; het, heterogeneity test.

Many potential risk factors were strongly associated with 14-day mortality as expected (Table 1; Supplementary Material). CDI cases, particularly those from clade 2 (PCR ribotype 027/ST 1), were older and generally had more of these risk factors. However, variations in 14-day mortality across *C. difficile* clades remained after adjustment (*P* < .0001; Figure 3). Strong evidence of higher mortality after clade 5 (PCR ribotype 078) vs clade 1 CDI (*P* = .001) and after clade 2 (PCR ribotype 027) vs clade 1 CDI (*P* = .002) persisted, with a trend toward higher mortality with clade 5 vs clade 2 CDI (*P* = .09). Further, although clades 3 and 5 are genetically similar in several pathogenicity locus genes [10], mortality differed significantly between clade 5 vs clade 3 CDI (*P* = .03). Within clade 1, adjusted 14-day mortality risks remained lower in ST 44 (hazard ratio [HR], 0.31 vs other clade 1; 95% CI, .10–.98; interaction *P* = .05). After adjustment, 14-day mortality decreased year-on-year from 2006 to 2011 in EIA-positive cases (HR per year, 0.88; 95% CI, .80–.96) but not EIA-negative controls (HR, 1.03; 95% CI, .99–1.07; interaction *P* = .002), with no evidence of differential effects in clade 2 (*P* = .91).

**Figure 4.**
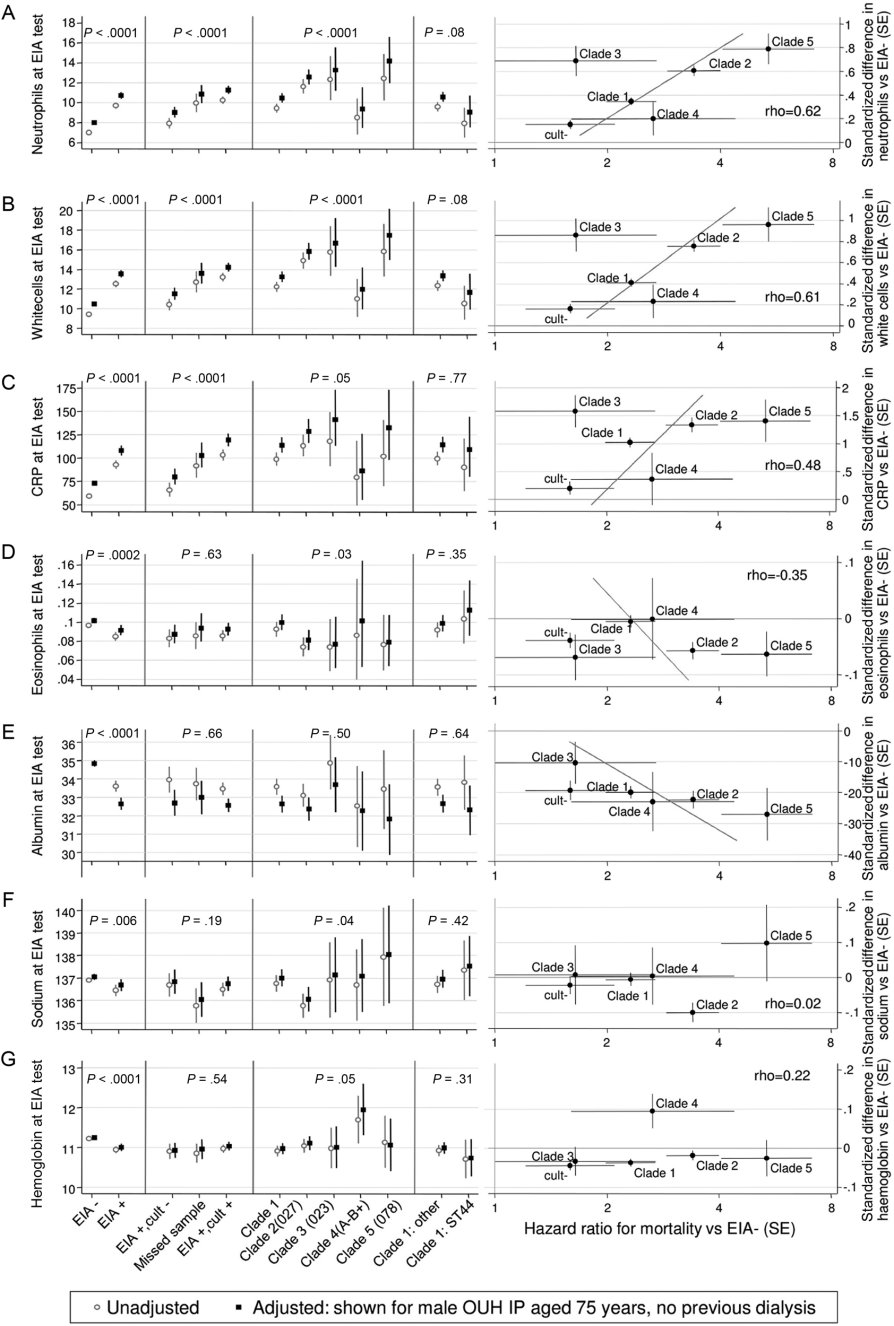
Variation in 7 biomarkers at diagnosis according to *Clostridium difficile* clade and association with mortality. *A*, Neutrophils (×10^9^/L). *B*, White cell count (×10^9^/L). *C*, C-reactive protein (mg/L). *D*, Eosinophils (×10^9^/L). *E*, Albumin (g/dL). *F*, Sodium (mmol/L). *G*, Hemoglobin (g/dL). For each biomarker, left-hand panels show mean (95% confidence interval) values at sample collection for enzyme immunoassay (EIA)–negative controls vs EIA-positive cases; then subdividing EIA-positive cases into culture-negative, not cultured, and culture-positive cases; then subdividing culture-positive cases by clade and comparing sequence type (ST) 44 vs other STs within clade 1; with *P* values testing for heterogeneity across each group. Means are calculated on BoxCox-transformed values and back-transformed for presentation (see Supplementary Methods). For each clade and EIA-positive/culture-negative cases, the right-hand panels plot the standardized adjusted mean difference vs EIA-negative controls from the left-hand panel (on the BoxCox-transformed scale,±standard error) against the adjusted hazard ratio for mortality vs EIA-negative controls from Table 1. The correlation, ρ, between biomarker and mortality risk excesses was estimated using multivariable random effects meta-analysis (see Supplementary Methods). Diagonal lines show the line of best fit (ie, the best prediction of excess mortality for any given excess in biomarkers compared with EIA-negative controls). If differences in biomarkers across clades completely explained mortality differences (ie, the biomarker was a perfect surrogate for mortality), all the points would lie on the diagonal line. The closer the points are to the diagonal line, the stronger the relationship between biomarker differences and excess mortality risks. Points lying far from the diagonal line indicate a mismatch, either high excess mortality with little difference in biomarkers from EIA-negative controls or vice versa. Abbreviations: CRP, C-reactive protein; cult, culture; EIA, enzyme immunoassay; OUH, Oxford University Hospitals; SE, standard error.

Variation in biomarkers at CDI diagnosis across clades and associations between excess biomarkers and excess mortality risks broadly followed three patterns. There was strong evidence for higher neutrophils/WBC in EIA-positive cases vs EIA-negative controls and in clades 2, 3, and 5 vs 1 (all *P* < .01) (Figure 4*A* and 4*B*; Supplementary Table 1). In clade 1–5 CDI cases, 31%, 46%, 48%, 21%, and 50%, respectively, had WBC > 15 × 10^9^/L (*P* < .0001) vs 15% in EIA-negative controls. Excess neutrophils/WBC and excess mortality risks were strongly associated across clades (rho = 0.6). However, clade 3 appeared dissimilar to other clades, with significantly higher neutrophil/WBC vs clade 1, similar to clades 2 (PCR ribotype 027/ST 1) and 5 (PCR ribotype 078/ST 11), despite significantly lower mortality. Variation across clades was similar, but slightly weaker, for CRP (*P* = .05) and eosinophils (*P* = .03; Figure 4*C* and 4*D*), with more severe (higher) CRP and (lower) eosinophils in clades 3 and 5. Associations between excess biomarker and mortality risks were also weaker (rho = 0.48, −0.35, respectively). At CDI diagnosis, albumin was significantly lower (Figure 4*E*) and platelets significantly higher (Supplementary Figure 1*H*) in EIA-positive cases vs EIA-negative controls (*P* < .0001), but there was no evidence of clade-specific differences (*P* > .50). In clades 1–5, 8%, 7%, 4%, 5%, and 15%, respectively, had albumin < 25 g/dL (*P* = .53) vs 5% in EIA-negative controls. However, excess mortality risks tracked reasonably closely with greater albumin reductions vs EIA-negative controls, suggesting that greater patient-level variation may have reduced power.

Serum sodium was slightly but significantly lower in EIA-positive cases vs EIA-negative controls (*P* = .006) and in clade 2 (Figure 4*F*). Although clades 2 and 5 had highest mortality, if anything, sodium was increased in clade 5 CDI (*P* = .08 vs clade 2), leading to no overall association between differences in sodium and excess mortality risks across the different clades (rho = 0.02). Hemoglobin was significantly lower in EIA-positive cases vs EIA-negative controls (*P* < .0001; Figure 4*G*), but clade-specific variation was restricted to higher hemoglobin in clade 4 (*P* = .05), with little association with excess mortality (rho = 0.22). Qualitatively, variation across clades in alanine aminotransferase (ALT), creatinine, estimated glomerular filtration rate [23, 24], and serum potassium was similar to hemoglobin (Supplementary Figure 1, *I*–*L*). No clear associations were evident for urea or alkaline phosphatase (Supplementary Figure 1*N* and 1*O*).

**Figure 5.**
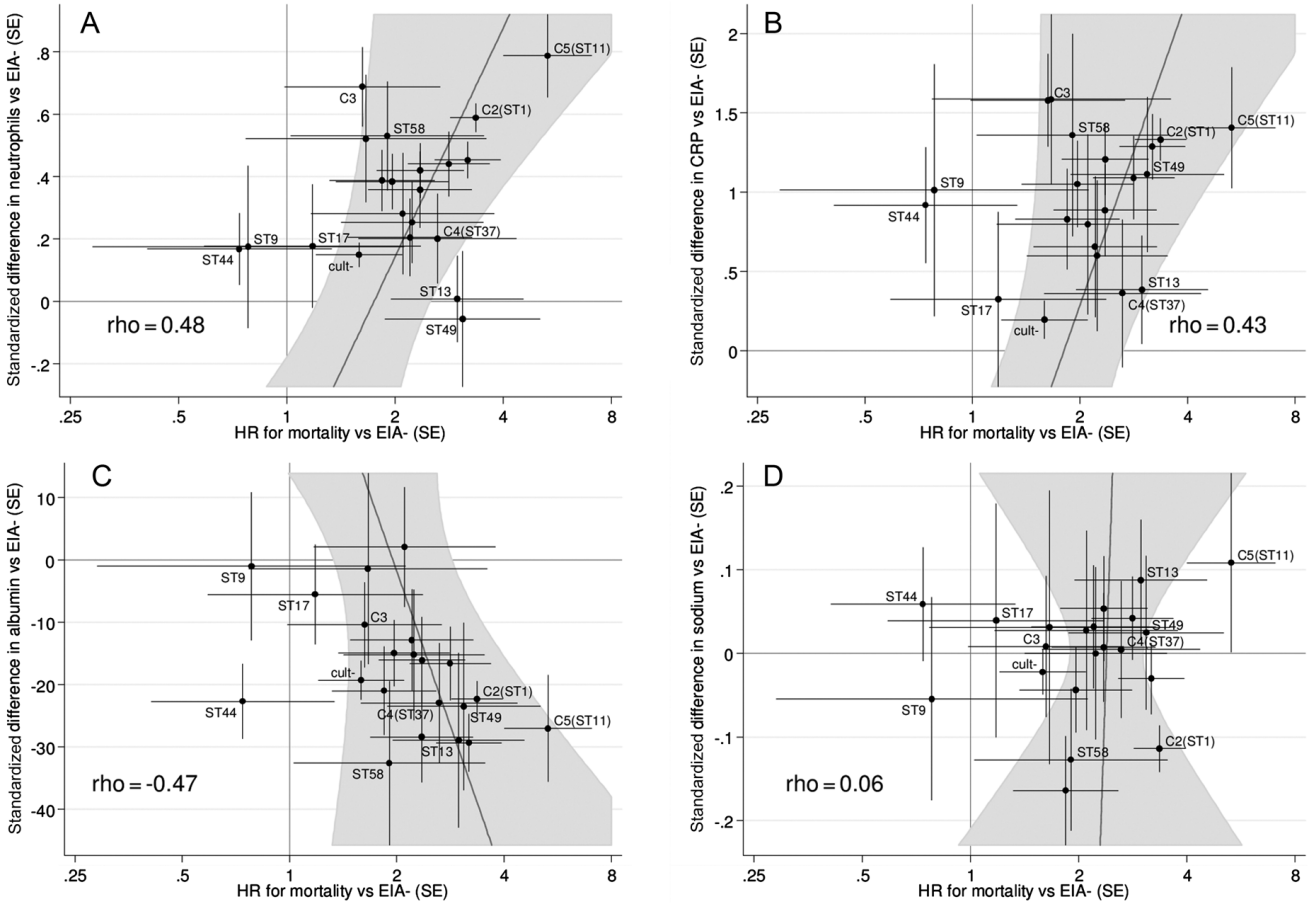
Impact of *Clostridium difficile* clade and individual sequence type (ST) on biomarkers compared with mortality. *A*, Neutrophils (×10^9^/L). *B*, C-reactive protein (mg/L). *C*, Albumin (g/dL). *D*, Sodium (mmol/L)*.* For clades 2–5 (labelled C2, C3, C4, C5) and each clade 1 ST with >20 isolates, the panels plot the standardized adjusted mean difference vs enzyme immunoassay (EIA)–negative controls (on the BoxCox-transformed scale,±standard error) against the hazard ratio for mortality vs EIA-negative controls, adjusted as in Table 1. The correlation, ρ, between biomarker and mortality risk excesses across STs/clades was estimated using multivariable random effects meta-analysis (see Supplementary Methods). Diagonal lines show the line of best fit (ie, the best prediction of excess mortality for any given excess in biomarkers compared with EIA-negative controls), together with a 95% credibility region indicated by the shaded region. If a biomarker was a perfect surrogate for mortality (ie, differences in biomarkers across STs/clades completely explained mortality differences), all the points would lie on the diagonal line. The closer the points are to the diagonal line, the stronger the relationship between biomarker differences and excess mortality risks. Points lying far from the diagonal line indicate a mismatch, either high excess mortality with little difference in biomarkers from EIA-negative controls or vice versa. All clade 1 STs lying outside the 95% credibility region on any of the 4 panels are labelled on each panel; ST 58, which had high mortality in [6], is also labelled. Abbreviations: CRP, C-reactive protein; cult, culture; EIA, enzyme immunoassay; HR, hazard ratio; SE, standard error; ST, sequence type.

Comparing associations individually for clade 1 STs (Figure 5) supported the partial surrogacy of differences in neutrophils/WBC (rho = 0.48), CRP (rho = 0.43), and eosinophils (rho = −0.45) for excess mortality risk but suggested a stronger relationship with albumin (rho = −0.47). Lack of association for other biomarker changes remained (eg, sodium rho = 0.06; Figure 5*D*). ST 44 was an outlier, with significantly lower albumin but similar neutrophils/CRP and mortality risk to EIA-negative controls.

Lastly, we estimated how much of the variation in *C. difficile* clade-associated mortality risk was related to observed biomarker differences. As expected given large numbers, all biomarkers except ALT independently predicted 14-day mortality in addition to Table 1 factors (Supplementary Table 2). However, association strength varied substantially, with albumin, urea, eosinophils, sodium, and CRP most strongly (and creatinine/estimated glomerular filtration rate most weakly) related to mortality. Adjusting for baseline biomarkers explained 41%, 32%, and 37% of the increased mortality due to clades 1, 2, and 5, respectively (Figure 3). However, even after adjusting for these biomarker differences across *C. difficile* clades (Figure 4), significant mortality risk variation by clade remained (*P* = .03), with significantly higher mortality persisting in clade 2 (PCR ribotype 027) vs clade 1 (*P* = .01) CDIs.

## DISCUSSION

In the largest population-based study of genotype and CDI severity to date, we have exhaustively investigated the relationships between strain types, biomarkers, other risk factors, and mortality. We have demonstrated unequivocally that PCR ribotype 027/NAP1/BI/ST 1 (clade 2) strains have been, and continue to be, associated with greater attributable mortality. This excess risk persists even after adjusting for large differences in severity biomarkers. Further, PCR ribotype 078 (clade 5) CDI has attributable mortality at least as great as PCR ribotype 027/ST 1, in agreement with 1 previous study [13] but in contrast with another [6]. Although PCR ribotype 078/clade 5 strains are currently present at low frequency, prospective surveillance demonstrates their continued expansion [25]; ongoing monitoring therefore remains essential.

Comprehensive simultaneous characterization of the impact of different *C. difficile* strains on biomarkers and mortality, not previously described to our knowledge, has enabled us to show that strain-type-specific excess mortality risk correlates most closely with strain-type-specific changes in inflammatory biomarkers. Conceptually the framework behind these analyses is similar to that for assessing surrogacy of intermediate for clinical outcomes (eg, blood pressure for cerebrovascular disease) [26]. Some biomarkers, notably renal-related biomarkers (creatinine, eGFR), were prognostic for mortality but did not vary significantly across CDI cases/controls or clades (ie, were acting independently of CDI). Others were prognostic and differed significantly between CDI cases and EIA-negative controls but not across clades. The most prognostic marker, albumin, fell into this category, possibly because of large variability. Biomarkers in the most interesting group, particularly neutrophils/WBC, CRP, and eosinophils, were prognostic and demonstrated evidence of partial surrogacy (ie, greater differences in baseline biomarkers between clades translated into greater differences in 14-day mortality). This has 2 consequences: First, quantitative traits like these biomarkers may provide greater power than time-to-event outcomes to detect effects of polymorphisms in genome-wide association studies. Second, surrogate markers indicate causal mechanisms of bacterial pathogenesis and may identify future therapeutic areas for investigation. Our results implicate inflammatory pathways as the major influence on poor outcome after CDI.

Although we found strong associations between strain-specific biomarkers and mortality overall, we also discovered intriguing exceptions that, as exploratory findings, may indicate important areas for future investigation. Specific genotypes within the large, heterogenous clade 1, notably ST 44, had particularly low 14-day mortality in post hoc analyses. Although ST 44 differs by only 1 nucleotide on MLST from ST 10, respective 14-day mortality was 3% and 11%, the latter typical of clade 1 overall (12%). However, both STs are consistently identified as PCR ribotype 015 [10]. They differ by >1500 single nucleotide polymorphisms across the genome [19] and may also differ in their accessory genomes, suggesting possible areas for future study. In contrast, our data suggest ST 49 (PCR ribotype 014) could be a more severe clade 1 genotype; this is an emergent clone in the United Kingdom [25] and should be monitored closely. Another intriguing finding is the major disconnect between the impact of clade 3 CDI on neutrophils/WBC/CRP and mortality. Similarities between clades 3 and 5 in severity biomarkers might be expected, as the receptor-binding domain of their pathogenicity locus *tcdB* gene (encoding one of the major known clostridial toxins) is highly genetically similar and their *tcdC* sequences share the same protein-truncating nucleotide substitution [10]. The latter is phenotypically equivalent to the single nucleotide deletion in the clade 2/PCR ribotype 027 *tcdC*, which causes a protein-truncating frameshift [10] and possibly leads to hypervirulence through increased toxin expression [27, 28] (although recent studies have questioned this [29]). Clades 2, 3, and 5 are also binary toxin positive [10] (in contrast with clades 1 and 4). However, the substantially lower mortality in clade 3 vs clade 5 highlights the importance of other, as yet undetermined, virulence or host factors to clinical outcomes [30] and suggests that increased toxin production alone in PCR ribotype 078 cannot account for its virulence.

Overall, we found 30%–40% of differences in mortality risk between strains were due to differences in biomarkers at diagnosis. However, in contrast with a recent much smaller study [31], even after adjusting for biomarker differences (and other factors) significant mortality differences remained across clades; this suggests that further microbial virulence determinants remain to be identified. Of note, the biomarker-adjusted effects of strain (reported in [31]) adjust away any effect of strain on outcome mediated through biomarkers, effects that we show to be substantial (Figure 4).

Our study has some limitations. The EIA assay used for case ascertainment has suboptimal sensitivity (91.7% in [32]), similar to other toxin EIAs [32, 33]. However, because of widespread concerns about sensitivity, for most of the study (through December 2009), multiple diarrheal samples were submitted from each patient, simultaneously or serially (500–1100 EIA tests performed monthly), reducing the chance of completely missing symptomatic CDI. One consequence is that we almost certainly identified false positives, perhaps explaining some EIA-positive/culture-negative cases [34]. To reduce the impact of false negatives, our controls only included EIA-negative tests >21 days before the first EIA positive result. During the study, there were 9.2 EIA-positive CDIs/10 000 overnight stays in inpatients, compatible with the 3.8–9.5 EIA-positive CDIs/10 000 overnight stays typical in endemic settings [35]. Overall, 14-day mortality attributable to EIA-positive CDI was 7.7%, similar to the 8% in a meta-analysis of 10 975 cases from 27 studies after 2000 [36] and 11% in another large study [37], also suggesting generalizability. By necessity, analyses were limited to available electronic data, which did not include previous/concomitant antibiotics, specific comorbid conditions, or causes of death. Although antibiotics are undoubtedly critical for developing CDI, given the lack of impact of adjusting for other important risk factors on strain–mortality associations, it is plausible that further adjustments would have had little further effect. Although theoretically *C. difficile*–related deaths should provide a more accurate measure of attributable mortality, practically attributing causes is subjective and usually unaudited. In contrast, all-cause mortality is objective, and differences in early mortality between EIA-positive cases vs EIA-negative diarrhea controls should be directly or indirectly CDI related. Although previous studies have considered 30-day mortality [5], reasonable reinfection rates between 14–30 days [38] influenced our prespecified choice of primary outcome. However, strain differences were similar at 30 days, and survival curves were parallel subsequently (Figure 2).

Our study also has important strengths. First is its comprehensive scope, including cases from an entire region over almost 5 years, including 3 hospitals providing acute services and numerous secondary/primary care providers. Second, it included 1893 EIA-positive/culture-positive strain-typed cases, approximately double the largest previous studies (n = 1008 [5]; n = 715 [13]). Study size becomes increasingly important when exploring differences between strains; 700–800 cases are needed to detect an 8% absolute mortality increase (as observed between clade 1 vs clade 2) with 80% power. Inadequate power therefore likely explains why smaller studies failed to identify associations between PCR ribotype 027 and severe outcomes (eg, n = 123 [7]; n = 128 [39]; n = 236 [40]). We were also able to compare strains at the clade/ST level, whereas most previous studies have only compared 027 vs non-027 strains [5], pooling 4 heterogeneous clades. We were unable to confirm previous reports [6] of poorer outcomes with PCR ribotypes 018 (ST 17 [10]) and 056 (ST 34/58 [10]), although longer-term mortality was similar in clade 4 (PCR ribotype 017/ST 37) and clade 2 (PCR ribotype 027/ST 1) as previously reported [11]. Our data confirm that the lack of the large clostridial toxin A (*tcdA*) in these clade 4 cases does not lead to less severe outcomes. We did not find any evidence of greater year-on-year mortality reductions in PCR ribotype 027/ST 1 (clade 2) compared with other clades [39], suggesting overall improvements in outcome are more likely due to better patient management than strain effects. The other mortality risk factors we identified broadly agree with previous studies [16], mostly reflecting disease severity or subsequent management; however, unlike previous studies, we have adjusted for the potential confounding due to bacterial type.

In summary, MLST demonstrates that strain predicts mortality and severity biomarkers at both clade and individual sequence-type level. For patient monitoring, neutrophils/WBC, CRP, and albumin are the key *C. difficile*–associated biomarkers that are highly prognostic for short-term mortality and also partial surrogates (with the possible exception of clade 3). For surveillance, PCR ribotype 078/ST 11 (clade 5) is associated with severe CDI, and its prevalence provides an important context for hospital mortality data [25]. Lastly, our study demonstrates the power from integrating large electronic databases with molecular sequence-based typing. Using whole-genome sequencing, approximately 85% of an approximately 4.3-Mb reference *C. difficile* genome can be called using standard mapping [19], providing unparalleled resolution to investigate severity determinants compared with the 7.4-kb MLST sequence used here. Unexpected differences in strains appearing highly similar by MLST and in biomarker vs mortality relationships hint at the advances that pathogen whole-genome association studies will provide in our understanding of bacterial pathogenesis over the next decade.

## Supplementary Data

Supplementary materials are available at *Clinical Infectious Diseases* online (http://cid.oxfordjournals.org/). Supplementary materials consist of data provided by the author that are published to benefit the reader. The posted materials are not copyedited. The contents of all supplementary data are the sole responsibility of the authors. Questions or messages regarding errors should be addressed to the author.

Supplementary Data
