# Supplementary material for: Relationship Between Bacterial Strain Type, Host Biomarkers, and Mortality in Clostridium difficile Infection
Source: Clin Infect Dis. 2013 Mar 5;56(11):1589–600. doi: 10.1093/cid/cit127 (PMC3641870; doi:10.1093/cid/cit127)
Supplement: Supplementary Data [file supp_56_11_1589__index.html]

Relationship Between Bacterial Strain Type, Host Biomarkers and Mortality in Clostridium difficile Infection — Relationship Between Bacterial Strain Type, Host Biomarkers, and Mortality in Clostridium difficile Infection — Relationship Between Bacterial Strain Type, Host Biomarkers, and Mortality in Clostridium difficile Infection — Supplementary Data 

# Relationship Between Bacterial Strain Type, Host Biomarkers, and Mortality in *Clostridium difficile* Infection

## Supplementary Data

Supplementary Data

**Files in this Data Supplement:**

- Supplementary Data - Doc file
