# Supplementary material for: Relationship Between Bacterial Strain Type, Host Biomarkers, and Mortality in Clostridium difficile Infection
Source: Clin Infect Dis. 2013 Mar 5;56(11):1589–600. doi: 10.1093/cid/cit127 (PMC3641870; doi:10.1093/cid/cit127)
Supplement: Supplementary Data [file supp_cit127_cit127supp.doc]

**SUPPLEMENTARY MATERIAL**

**Statistical Methods**

**Supplementary Results**

**Supplementary Table 1 Variation in biomarkers at CDI diagnosis**

**Supplementary Table 2 Impact of biomarkers at CDI diagnosis on 14-day mortality**

**Supplementary Figure 1 Variation in 9 further biomarkers at diagnosis according to *C. difficile* clade, and association with mortality**(h) Platelets (10^9^/l)
(i) ALT (IU)
(j) Creatinine (mmol/l)
(k) eGFR – MDRD (ml/min)
(l) eGFR – CKEPI (ml/min)
(m) Potassium (mmol/l)
(n) Urea (mmol/l)
(o) Alkaline Phosphatase (IU)

Footnote For each biomarker, left-hand panels show mean (95% confidence interval, CI) values at sample collection for EIA-negatives vs positives; then subdividing EIA-positives into culture-negative, not cultured, culture-positive; then subdividing culture-positives by clade, and comparing ST44 vs other STs within clade 1; with p-values testing for heterogeneity across each group. Means are calculated on BoxCox-transformed values and back-transformed for presentation (see Supplementary Methods). For each clade and EIA-positive-culture-negatives, the right-hand panels plot the standardised adjusted mean difference versus EIA-negatives from the left-hand panel (on the BoxCox-transformed scale, ±standard error, SE) against the adjusted hazard ratio for mortality versus EIA-negatives from Table 1. The correlation, , between biomarker and mortality risk excesses was estimated using multivariate random effects meta-analysis (see Supplementary Methods). Diagonal lines show the line of best fit, ie the best prediction of excess mortality for any given excess in biomarkers compared to EIA-negatives. If differences in biomarkers across clades completely explained mortality differences (ie the biomarker was a perfect surrogate for mortality) all the points would lie on the diagonal line. The closer the points are to the diagonal line, the stronger the relationship between biomarker differences and excess mortality risks. Points lying far from the diagonal line indicate a mismatch, either high excess mortality with little difference in biomarkers from EIA-negatives, or vice versa.

**STATISTICAL METHODS**

The choice of 14 days for the primary mortality outcome was determined by (i) separate analyses of strain switching demonstrating that the odds of re-infection vs recurrence increased >5-fold over the first 14 days^1^, meaning that this initial 14-day period was least likely to be affected by subsequent acquisition of a new infecting strain (ii) the 14 day vancomycin treatment course, meaning that this initial 14-day period represented initial treatment response rather than any effect of subsequent treatment (iii) to increase the likelihood that all-cause mortality was *C. difficile*-related, as the age of patients included meant that non-*C. difficile* related mortality would be expected over the longer-term and (iv) to reduce the impact of non-proportional hazards, since the effect of factors at the time a sample was taken for testing would be expected to wane over the longer term. In terms of the primary exposure of interest, *C. difficile* clade, we^1^ have previously found relatively low rates of mixed infections (3-7%), similarly to previous studies. We therefore assumed that where only one isolate had been MLST-ed (the majority of cases), that the most prevalent infecting strain would both be most likely to be sequenced and to be the causative agent, that is that any low-frequency co-carried strains had less impact on mortality. In 17 (0.6%) cases where multiple strains were isolated on the same day or within 14 days of an index test, the most common strain in the population was used to categorise the strain in analysis.

In 93.3% of CDIs/controls, vital status was known 14 days after sample collection (eg from subsequent admission/outpatient visit/sample in IORD, or death during admission or from routine updates from the NHS National Tracing Service).^2^ A further 3.5% of participants were known to be alive 1-13 days after sample collection, and for 3.2% the last available information was on the sample collection date; as expected, these CDIs/controls were more likely to have been tested outside hospital and in the most recent time period. The 6.7% patients known in IORD to be alive 0-13 days after sample collection were assumed alive 14 days after sample collection, assuming data from the NHS Tracing Service is complete^2^. Sensitivity analyses censoring these patients at the date of last information were similar (data not shown).

Multivariable Cox models for mortality (as a time-to-event variable) considered factors a priori considered to affect mortality risk, and those previously associated with *C. difficile* mortality, namely demographics, sample characteristics, previous hospital exposure, and previous healthcare-associated infections. Previous hospital exposures and healthcare-associated infections were defined on the basis of information strictly preceding the admission date for inpatient CDI, to ensure they acted as potential confounders rather than possible effect mediators. Some factors at sample collection could conceivably act as either confounder or effect mediator; confounding could arise if, for example, particular strains were transmitted amongst more similar patients in specific areas of the hospital, and effect mediation could arise if, for example, those infected with more severe strains were more likely to be admitted as inpatients. As it was impossible to exclude the possibility of sample characteristics being potential confounder, they were included in the main multivariable model. Baseline biomarkers, which represent a response to infection, were considered in separate adjusted models, because they are highly likely to be mediators rather than confounders, ie on the causal pathway between CDI and mortality.

Independent risk factors for 14-day mortality were identified using backwards elimination with the Akaike Information Criterion (AIC)^3^. Pairwise interactions between all terms in the multivariable model were then investigated using forward selection (also with AIC). We also tested whether any ST within clade 1 was associated with a significantly different impact on mortality to other clade 1 STs using forward selection. Interactions resulting in a reduction in AIC were retained in the model. Selection procedures based on AIC were used to ensure that all possible confounders of any mortality effects in the small numbers of CDI cases from clades 3-5 were included in adjustments. Final multivariable models were similar using the same methods on 90-day mortality, with somewhat more pronounced mortality risks in clade 4 vs 1 as demonstrated in Figure 2 (detailed data not shown). Similar results were also obtained using only the first ever EIA test per person and restricting to inpatients only (not shown).

Baseline biomarker values were defined as the closest measurement within [-3,+1] calendar days of the sample collection date. All laboratory haematology and biochemistry biomarkers that were available at baseline on >50% CDI cases overall were evaluated in analyses; results from only lymphocytes are not presented as differences between clades, and impact of adjustment on mortality, was essentially identical to neutrophil counts (ie lymphocytes added no extra information). Across the 15 different biomarkers evaluated and presented, baseline values were available for 82-94% of samples from inpatients, 28-50% samples from other hospitals/outpatients/ER/day cases and 6-18% samples from primary care. For example, biomarkers that were not evaluated due to low numbers included phosphate, AST, chloride, and bicarbonate (available on <20% at baseline), Estimated Glomerular Filtration Rate (eGFR) was calculated using both the Modification of Diet in Renal Disease (MDRD)^4^ and Chronic Kidney Disease Epidemiology Collaboration (CKD-EPI)^5^ formulae. Biomarkers were BoxCox transformed to improve normality, and results from normal linear regression models fitted on transformed values were then back-transformed for presentation of average values by clade (Figures 4, 5, Supplementary Figure 1). For C-reactive-protein, 25% of recorded values were above the assay threshold (>160 mg/l), and so interval normal regression, which allows for this threshold, was used instead of standard linear regression^6^. Differences between *C. difficile* clades in biomarkers were similar not adjusting for other factors; adjusting for sex, age, sampling location and previous dialysis only; and adjusting for all factors considered for the mortality analysis (data not shown).

From these models each *C. difficile* clade was associated with an excess mortality risk versus EIA-negatives (log hazard ratio, HR) and a difference in standardised baseline biomarkers versus EIA-negatives. If differences in biomarkers across clades completely explained mortality differences (ie the biomarker was a perfect surrogate for mortality), then there would be perfect correlation between these two estimates across the 5 clades and EIA-positive-culture-negatives. However, this correlation cannot be estimated using standard methods, because it is a correlation between two sets of estimates, rather than two sets of observations. Instead it was estimated using multivariate random effects meta-analysis^7^ with Riley’s overall correlation model^8^ treating clades as independent “studies” (given the large number of EIA-negative controls and small correlations between effect estimates across clades (|ρ|<0.1) (mvmeta in Stata)). The best fitting linear relationship between the log(HR) and the Boxcox-standardised baseline biomarker differences was estimated using the method of Daniels and Hughes^9^ implemented in WinBugs, setting the correlation parameter to the estimate from the multivariate random effects meta-analysis. Results were similar varying the correlation parameter across plausible ranges as suggested^9^ (data not shown).

To estimate the impact of baseline biomarkers on 14-day mortality, missing biomarker values in the subset of 20728 (68%) patients with ≥1 observed baseline biomarker (2022 (74%) CDI cases, 18706 (68%) controls) were imputed 25 times using chained estimating equations^10, 11^. The numbers of observed values for each biomarker are given in Supplementary Table 1: only 15055 (50%) patients had values observed for all 15 biomarkers (1508 (55%) CDI cases, 13547 (49%) controls). As recommended^12^, imputations were done on the BoxCox transformed variables, including all the Table 1 cofactors, allowing non-linearity in all continuous factors with natural cubic splines with three knots at the 10^th^, 50^th^ and 90^th^ percentiles^13^ and including log(survival time) and the censoring indicator to 14 days in imputation models. Cox proportional hazards models for 14-day mortality initially allowed for non-linearity in the effects of baseline biomarkers using natural cubic splines^13^. Where significant non-linearity was identified (p<0.0001), this was approximated with piecewise linear trajectories with changepoints chosen based on the fit of the natural cubic spline (see Supplementary Table 2). Effect sizes were approximately similar restricting Cox models to the 15055 (50%) patients who had all 15 biomarkers observed (“complete cases”, not shown).

Stata 11.2 and Winbugs 1.4.3 were used for analyses, conducted by ASW.

**SUPPLEMENTARY RESULTS**

Independently, 14-day mortality was higher in men and those with previous dialysis/chemotherapy (Table 1). Risk increased with increasing age and total previous time admitted to OUH, and decreased with increasing time since last discharge. Mortality risks were higher following samples taken in emergency (vs elective) inpatient admissions, and lower in those with samples taken outside OUH (even more so for EIA-negative tests), EIA tests that had not originally been requested by the clinician in those ≥65 years (mild diarrhoea), and those with more recent EIA-negative tests – all likely related to CDI severity. Lower 14-day mortality risks were observed in those with previous GI admission, possibly representing incidental detection of *C. difficile* in diarrhoeal samples being tested for alternative causes. 14-day mortality risk was higher in medical inpatients with EIA-negative or EIA-positive-culture-negative tests compared to medical inpatients with EIA-positive-culture-positive tests and surgical inpatients, possibly because medical inpatients with EIA-negative or culture-negative tests were more likely to have diarrhoea due to antibiotics for different underlying infections (eg pneumonia/urosepsis). Inpatient risks were highest for tests taken on the day of admission and ≥10 days later, dropping sharply after day 0 then rising gradually with increasing length of current stay (non-linearity p<0.0001). Adjusting for other factors, patients without any previous OUH admissions also had higher mortality risks, possibly due to delays seeking healthcare or transfer of critically unwell out-of-area patients to the OUH for specialist care. Interestingly, for the same total amount of previous time in OUH, mortality risks were lower in those who had spent this time across more previous admissions, and highest in those who had spent this time in one single long admission. This could reflect increased co-morbidity associated with long single admissions, or a "survivor" effect in those with multiple hospital discharges. If anything, 14-day mortality risks were lower in those with previous EIA-positives (adjusted hazard ratio, aHR=0.84 (95% CI 0.66-1.08) p=0.18), with no evidence of variation according to whether the same vs. different STs were identified (interaction p=0.67).

We found no evidence that the increasing mortality risk with age was more (or less) pronounced in EIA-positives (interaction p=0.13), *C. difficile* culture-positives (p=0.21) or clade 2 (PCR-ribotype-027) (p=0.19). After adjustment (Table 1), 14-day mortality decreased year-on-year from 2006-2011 in EIA-positives (HR per year=0.88 (0.80-0.96)) but not EIA-negatives (HR=1.03 (0.99-1.07), interaction p=0.002), with no evidence that these calendar trends varied in clade 2 (p=0.91). No other factor effects varied by type of EIA-positive (p>0.10) or clade 2 (p>0.18).

**SUPPLEMENTARY MATERIAL REFERENCES**

1. Eyre DW, Walker AS, Griffiths D, Wilcox MH, Wyllie DH, Dingle KE, et al. Clostridium difficile mixed infection and re-infection. Journal of clinical microbiology. 2011;(in press).

2. Porter K, Johnson AM, Phillips AN, Darbyshire JH. The practical significance of potential biases in estimates of the AIDS incubation period distribution in the UK register of HIV seroconverters. AIDS (London, England). 1999 Oct 1;13(14):1943-51.

3. Burnham KP, Anderson DR. Model selection and multimodel inference (Second Edition). New York: Springer; 2002.

4. Levey AS, Bosch JP, Lewis JB, Greene T, Rogers N, Roth D. A more accurate method to estimate glomerular filtration rate from serum creatinine: a new prediction equation. Modification of Diet in Renal Disease Study Group. Ann Intern Med. 1999 Mar 16;130(6):461-70.

5. Levey AS, Stevens LA, Schmid CH, Zhang YL, Castro AF, 3rd, Feldman HI, et al. A new equation to estimate glomerular filtration rate. Ann Intern Med. 2009 May 5;150(9):604-12.

6. Amemiya T. Regression analysis when the dependent variable is truncated normal. Econometrica. 1973;41:997–1016.

7. White IR. Multivariate random-effects meta-regression: Updates to mvmeta. The Stata Journal,. 2011;11(2):255-70.

8. Riley RD, Thompson JR, Abrams KR. An alternative model for bivariate random-effects meta-analysis when the within-study correlations are unknown. Biostatistics. 2008 Jan;9(1):172-86.

9. Daniels MJ, Hughes MD. Meta-analysis for the evaluation of potential surrogate markers. Stat Med. 1997 Sep 15;16(17):1965-82.

10. van Buuren S, Boshuizen HC, Knook DL. Multiple imputation of missing blood pressure covariates in survival analysis. Statistics in Medicine. 1999;18:681-94.

11. Royston P. Multiple imputation of missing values: update of ice. Stata Journal. 2005;5:527-36.

12. Sterne JA, White IR, Carlin JB, Spratt M, Royston P, Kenward MG, et al. Multiple imputation for missing data in epidemiological and clinical research: potential and pitfalls. BMJ. 2009;338:b2393.

13. Hess KR. Assessing time-by-covariate interactions in proportional hazards regression models using cubic spline functions. Statistics in Medicine 1994;13:1045-62.

**Supplementary Table 1 Variation in biomarkers at CDI diagnosis**

|  | Number (%) with measurement at diagnosis | | Type * (evidence) | Level of biomarker varies** (p-value) by | | | | | Pairwise comparisons** | | | | |
| --- | --- | --- | --- | --- | --- | --- | --- | --- | --- | --- | --- | --- | --- |
|  | EIA negative (N=27550) | EIA positive (N=2745) |  | EIA-negative vs EIA-positive | within EIA-positives: by culture-negative, not retrieved, culture-positive | within culture-positives: by 5 *C difficile* clades | across clades 1, 2, 3, 5 | clade 4 vs rest | Clade  1 vs 2 | Clade  1 vs 5 | Clade  2 vs 5 | Clade  1 vs 3 | Clade 1: ST44 vs rest |
| Neutrophils | 18301 (66%) | 1982 (72%) | 1 (strong) | **<0.0001** | **<0.0001** | **<0.0001** | **<0.0001** | **<0.0001** | **<0.0001** | **0.0009** | 0.18 | **0.008** | **0.08** |
| White cells | 18366 (67%) | 1986 (72%) | 1 (strong) | **<0.0001** | **<0.0001** | **<0.0001** | **<0.0001** | **<0.0001** | **<0.0001** | **0.0009** | 0.23 | **0.005** | **0.08** |
| CRP | 16862 (61%) | 1860 (68%) | 1 (mod) | **<0.0001** | **<0.0001** | **0.05** | **0.08** | **0.05** | **0.05** | 0.32 | 0.86 | **0.06** | 0.77 |
| Eosinophils | 18269 (66%) | 1973 (72%) | 1 (mod) | **0.0002** | 0.63 | **0.03** | **0.01** | **0.03** | **0.004** | 0.16 | 0.89 | 0.12 | 0.35 |
| Albumin | 16228 (59%) | 1746 (64%) | 1 | **<0.0001** | 0.66 | 0.50 | 0.35 | 0.50 | 0.47 | 0.41 | 0.60 | 0.18 | 0.64 |
| Platelets | 18349 (67%) | 1983 (72%) | 1 | **<0.0001** | 0.18 | 0.60 | 0.50 | 0.60 | 0.29 | 0.54 | 0.83 | 0.40 | 0.24 |
| Alk. Phos | 15908 (58%) | 1700 (62%) | 1 | **<0.0001** | 0.22 | 0.62 | 0.46 | 0.62 | 0.43 | 0.21 | 0.35 | 0.33 | **0.06** |
| Urea | 16572 (60%) | 1841 (67%) | 1 | 0.32 | 0.12 | 0.13 | 0.11 | 0.13 | 0.90 | **0.05** | **0.05** | 0.16 | 0.68 |
| Sodium | 18360 (67%) | 1988 (72%) | 2 (mod) | **0.006** | 0.19 | **0.04** | **0.02** | **0.04** | **0.004** | 0.35 | **0.08** | 0.88 | 0.42 |
| Haemoglobin | 18367 (67%) | 1986 (72%) | 3 (mod) | **<0.0001** | 0.54 | **0.05** | 0.68 | **0.05** | 0.21 | 0.80 | 0.88 | 0.92 | 0.31 |
| ALT | 15799 (57%) | 1682 (61%) | 3 | **<0.0001** | **0.0001** | 0.40 | 0.69 | 0.40 | 0.27 | 0.85 | 0.57 | 0.84 | 0.22 |
| Potassium | 18359 (67%) | 1989 (72%) | 3 | **<0.0001** | 0.22 | 0.62 | 0.88 | 0.62 | 0.94 | 0.45 | 0.44 | 0.85 | 0.24 |
| Creatinine | 18346 (67%) | 1988 (72%) | 3 | 0.89 | 0.66 | 0.59 | 0.78 | 0.59 | 0.33 | 0.93 | 0.70 | 0.87 | 0.90 |
| eGFR (MDRD)† | 18346 (67%) | 1988 (72%) | 3 | 0.99 | 0.60 | 0.66 | 0.84 | 0.66 | 0.40 | 0.98 | 0.81 | 0.83 | 0.94 |
| eGFR (CK-EPI)‡ | 18346 (67%) | 1988 (72%) | 3 | 0.67 | 0.53 | 0.72 | 0.86 | 0.72 | 0.43 | 0.66 | 0.87 | 0.97 | 0.86 |

* type of pattern of changes in biomarkers across EIA-positive vs EIA-negative and clades, and relationship with mortality differences, see Figure 3 and Supplementary Figure 1. Statistical evidence supporting pattern of changes in brackets: otherwise grouping is based on visual inspection of Supplementary Figure 1.

** adjusted for sex, age, location of patient when sample taken, and previous dialysis.

† estimated glomerular filtration rate (eGFR) using the Modification of Diet in Renal Disease (MDRD) formula^4^

‡ estimated glomerular filtration rate (eGFR) using the Chronic Kidney Disease Epidemiology Collaboration (CK-EPI) formula^5^

Note: p values <0.10 indicated in bold. See Figure 3 and Supplementary Figure 1 for means in each group.

**Supplementary Table 2 Impact of biomarkers at CDI diagnosis** on 14-day mortality

| **Factor*** | **Unadjusted  univariable model** | **Adjusted  multivariable model**** |
| --- | --- | --- |
|  | HR (95% CI) p | HR (95% CI) p |
| Neutrophils: per 3x10^9^/l higher | 1.30 (1.27-1.33) 2x10^-129^ | 1.09 (1.06-1.12) 6x10^-9^ |
| White cells: per 3x10^9^/l higher | 1.23 (1.21-1.25) 5x10^-109^ | † |
| CRP: per 50 mg/l higher | 1.57 (1.50-1.63) 1x10^-88^ | 1.18 (1.12-1.24) 7x10^-11^ |
| Eosinophils: per 0.1x10^9^/l higher <0.2  per 0.1x10^9^/l higher >0.2 | 0.52 (0.49-0.56) 1x10^-73^  1.03 (0.97-1.09) | 0.72 (0.66-0.77) 6x10^-19^  0.97 (0.92-1.03) |
| Sodium per 3 mmol/l higher <136  per 3 mmol/l higher >136 | 0.72 (0.68-0.75) 1x10^-108^  1.47 (1.42-1.52) | 0.88 (0.83-0.93) 1x10^-16^  1.18 (1.14-1.23) |
| Haemoglobin per 1.5 g/dl higher <11  per 1.5 g/dl higher >11 | 0.75 (0.70-0.81) 1x10^-23^  0.92 (0.86-0.99) | † |
| ALT per 10 IU higher | 1.03 (1.02-1.03) 3x10^-12^ | ‡ |
| Alk. Phos per 100 IU higher <350  per 100 IU higher >350 | 1.54 (1.43-1.64) 1x10^-52^  1.03 (1.02-1.05) | 1.22 (1.13-1.31) 1x10^-10^  1.02 (1.00-1.04) |
| Potassium per 0.3 mmol/l higher <3.9  per 0.3 mmol/l higher >3.9 | 0.90 (0.86-0.95) 8x10^-60^  1.25 (1.22-1.29) | 0.94 (0.90-0.99) 2x10^-8^  1.11 (1.07-1.15) |
| Albumin per 5 g/dl higher | 0.61 (0.59-0.63) 1x10^-141^ | 0.74 (0.71-0.78) 2x10^-30^ |
| Platelets per 100x10^9^/l higher <300  per 100x10^9^/l higher >300 | 0.72 (0.67-0.76) 7x10^-25^  1.05 (0.99-1.11) | 0.79 (0.73-0.85) 2x10^-9^  1.01 (0.95-1.08) |
| Urea per 3 mmol/l higher <12  per 3 mmol/l higher >12 | 1.72 (1.62-1.81) 1x10^-174^  1.10 (1.08-1.12) | 1.35 (1.24-1.46) 3x10^-19^  1.08 (1.04-1.11) |
| Creatinine per 25 μmol/l higher <125  per 25 μmol/l higher >125 | 1.34 (1.27-1.41) 7x10^-53^  1.03 (1.02-1.04) | 0.76 (0.61-0.91) 0.002  0.96 (0.93-0.98) †† |
| eGFR (MDRD) per 20 ml/min higher <80  per 20 ml/min higher >80 | 0.64 (0.61-0.67) 2x10^-41^  1.12 (1.07-1.18) | † |
| eGFR (CK-EPI) per 20 ml/min higher <70 per 20 ml/min higher >70 | 0.61 (0.58-0.65) 2x10^-78^  0.87 (0.81-0.94) | 0.75 (0.59-0.91) 0.002  1.00 (0.81-1.20) |

* Increments for hazard ratios (HR) are approximately half the interquartile range, so that two biomarkers with a similar predictive effect across the variation observed in the study would have similar HRs. Non-linearity initially modelled using natural cubic splines^13^, then approximated with piecewise linear trajectories with the given changepoint chosen based on the fit of the natural cubic spline. All p<0.02 for heterogeneity in effects above and below the changepoint in adjusted model (heterogeneity p<0.0001 in univariable models).

** Adjusted for all biomarkers shown (as main effects only) and also adjusted for all factors in multivariable model in Table 2 (effects substantially weaker (p>0.15) for sample taken in primary care, negative if not an inpatient, mandatory EIA test (mild diarrhoea), never previously admitted, number of previous admissions >8h, previous dialysis, and previously admitted to a GI ward suggesting that these factors and severity biomarkers may have a common cause; other estimates similar suggesting effects are independent of biomarkers).

*** Heterogeneity within EIA-positives: by culture-negative, not retrieved, culture-positive.

† Not considered for full model: (i) white cell count collinear with neutrophils (Spearman rho=0.96) with less variation explained in univariable models, (ii) haemoglobin associated with albumin (Spearman rho=0.56) and including both in a multivariable model leads to over-correction and higher haemoglobin associated with increased risks of death for the same level of albumin, (iii) eGFR similar whether calculated by MDRD or CK-EPI (Spearman rho=0.99) with less variation explained by MDRD in univariable models.

‡ No independent effect of ALT at diagnosis adjusting for other factors (p=0.10).

†† Higher creatinine associated with decreased risk of death adjusted for other biomarkers (in contrast to increased risk of death univariably): likely reflecting lower risk of death in patients with normal weight compared to those malnourished. Reversal of association compared with univariable effect was apparent if either eGFR (MDRD or CKEPI) or urea were adjusted for with creatinine at sample colleciton. Weight was not available in the underlying electronic data sources.
